# Monte Carlo simulation of testing requirements for chronic wasting disease (CWD) surveillance via hunter-harvest in Illinois, USA

**DOI:** 10.1080/19336896.2026.2725518

**Published:** 2026-09-02

**Authors:** Jameson J. Mori, William M. Brown, Nelda A. Rivera, Daniel J. Skinner, Peter E. Schlichting, Jan E. Novakofski, Nohra E. Mateus-Pinilla

**Affiliations:** a Illinois Natural History Survey, University of Illinois Urbana-Champaign, Champaign, IL, USA; b Illinois Department of Natural Resources, Division of Wildlife Resources, Springfield, IL, USA; c Department of Animal Sciences, University of Illinois Urbana-Champaign, Champaign, IL, USA; d Department of Pathobiology, University of Illinois Urbana-Champaign, Urbana, IL, USA; e Department of Natural Resources & Environmental Sciences, University of Illinois Urbana-Champaign, Urbana, IL, USA

**Keywords:** Cervid, chronic wasting disease, CWD, management, surveillance, transmissible spongiform encephalopathy, TSE, wildlife

## Abstract

Managing chronic wasting disease (CWD) – a fatal, transmissible prion disease of cervids – has many challenges, such as determining the number of cervids to test to accurately estimate true prevalence with apparent prevalence. Options range from basic equations to complex techniques like agent-based modelling, but the former does not account for parameter uncertainty, and more detailed models may be less accessible for managers. Our study used Monte Carlo simulation to create a reference table showing the number of hunter-harvested deer to test to approximate herd true prevalence with a ± 2% margin of error and 95% confidence. A three-step process was simulated in which deer from the *total population* were harvested (*testable subpopulation*), and then a subset of those harvested deer were tested for CWD (*tested group*) with diagnostic testing error, with group assignment determined by random sampling. We evaluated true prevalences ranging from 0.5–20% and *testable subpopulation* sizes up to 5,000 deer from a total population of 20,000. An example from the reference table shows that detecting a 1% true prevalence in a *testable subpopulation* of 300 deer requires testing ≥280 deer, and that the only way to accurately detect a true prevalence of 0.5% is to test 600 out of 5,000 deer. Evaluating IDNR’s CWD surveillance efforts found that 16.14% of counties had adequate sample sizes in a given year and could detect true prevalences as low as 1%, showing that these guidelines are achievable.

## Introduction

Chronic wasting disease (CWD) is an invariably fatal neurodegenerative disease of cervids detected in wild and captive herds in the United States, Canada, Norway, Sweden, and Finland since its discovery in 1967 [1]. Cases of CWD in captive cervid farms in South Korea, linked to imports from Canada, have also been reported [1]. The U.S. state of Illinois has intensively managed CWD since fiscal year 2003 (between July 1 of one calendar year and June 30 of the next), and this has involved laboratory testing of deer that were hunted, killed in vehicle collisions, removed in population control efforts, or culled as part of the Illinois Department of Natural Resources (IDNR) CWD control program [2,3]. The majority of deer tested for CWD in Illinois come from hunter-harvest [4], which is usually the case in other U.S. states as well [5]. Testing is conducted either on the retropharyngeal lymph nodes or obex, and disease is diagnosed with either immunohistochemistry (IHC) or enzyme-linked immunosorbent assay (ELISA) followed by IHC [4]. Sample submission is sometimes mandatory, sometimes voluntary, depending on state policies and the location of harvest [5].

For disease surveillance efforts to accurately inform management decisions, it is necessary to know whether enough deer are being tested to ensure that the apparent prevalence (estimated prevalence from tested deer) accurately approximates the total population’s true prevalence (actual prevalence of disease in the population). Previous studies of sample size and CWD have evaluated the probability of detecting CWD by testing hunter-harvested deer [6,7]. Diefenbach, Rosenberry, and Boyd identified deer abundance, disease prevalence, diagnostic testing sensitivity and specificity, and the probability of sampling an individual deer as driving detection of CWD in adult deer, but assumed a perfect diagnostic test and only tested a 1% prevalence. Rees et al. considered how variables like spatiotemporal infection patterns, spatial scale of sampling, demographics of harvested deer, and landscape variables like agriculture and terrain ruggedness impact detection of newly emerging disease outbreaks. Walton et al. looked at how sample size estimates are impacted by fluctuations in disease prevalence and host population size and found that these changes can lead to bias and overconfidence in these estimates [8]. Walsh and Miller [9] designed a frequentist weighted surveillance system to detect CWD cases in locations without previous detections based on deer age, sex, and health, while Jenelle et al. [10] used a Bayesian approach to generate a weighted surveillance strategy based on deer sex and age to enable early detection of emerging CWD outbreaks. Joly et al. [11] considered the role of deer abundance, nonrandom spatial distribution of hunter-harvest and CWD, and the geographic extent of the outbreak in their development of a surveillance system that could detect a prevalence of 1% in deer >1 year old with a 99% probability. Belsare et al. utilized an agent-based model of CWD dynamics in a deer population developed by Belsare and Steward to estimate sample size requirements using spatiotemporally heterogeneous parameters. This model simulated the distribution of a deer population with a given disease prevalence and accounted for the impact of factors like deer density, sex ratios, population size and growth/reproduction, age composition, mortality, and between deer interactions to generate sample size recommendations that were less conservative that traditional sample size calculations [12,13].

While these complex models are important for understanding the epidemiology of CWD and tailoring management actions to a specific spatial extent, the results of many of these studies may not be immediately applicable to wildlife managers, who must use complex equations or associated software to apply the findings to their field conditions. These models also leave geographical areas not included in the model without much guidance and only report sample size recommendations for a limited number of true prevalences. Several models also focus on detecting emerging outbreaks and lack suggestions for surveillance in endemic areas. Samples are also usually drawn directly from a total population that is often assumed to be infinite, ignoring the fact that wildlife managers and hunters do not have access to the total population. Assuming access to the total population therefore overlooks a stage in the surveillance process that shapes the final group of deer from which the apparent prevalence is calculated.

The current absence of resources for wildlife managers that want more accurate sample size guidelines that account for uncertainty but cannot use more detailed models – either because they are too technical or do not apply to locations they are managing – highlights an opportunity to provide sample size recommendations in a user-friendly format (i.e. a table) that can serve as a stopgap until better options are created. To accomplish this, we developed a Monte Carlo simulation of the CWD surveillance process in the Illinois white-tailed deer (*Odocoileus virginianus*) herd to quantify how many deer should be tested for CWD in a single fiscal year to obtain an apparent prevalence that is within ±2% of the true prevalence for true prevalences between 0.5–20% and for a total population of up to 20,000 deer. A reference table was generated from the simulation results for wildlife managers that can be used to evaluate CWD surveillance efforts and resource allocation in endemic areas.

## Methods

### Overview of Monte Carlo simulation

Monte Carlo simulation is a modelling technique that allows explicit characterization of uncertainty by using distributions of possible parameter values, rather than single point estimates [14]. This simulation method works by randomly selecting one value from each input parameter’s distribution and entering those values into the model equation(s) to produce a single output [14]. That process is then repeated for a designated number of iterations until a representative distribution of the output is generated [14]. This final distribution can then be analysed to more fully understand the system being studied.

### Monte Carlo simulation of CWD surveillance sample size requirements

Monte Carlo simulation was conducted in R [15] to determine the number of hunter-harvested white-tailed deer (hereafter referred to as ‘deer’) that must be tested for CWD in a single fiscal year for the calculated apparent prevalence to be within ±2% of the true prevalence in ≥95% of simulation iterations. This margin of error (±2%) means that the simulation results are more applicable to CWD-endemic areas, not locations where the disease is emerging. The fiscal year is the period between July 1^st^ of one calendar year and June 30^th^ of the next and encompasses the breeding season in fall and the subsequent fawning in the spring, capturing a single reproductive cycle, as well as including both hunter-harvest (fall/winter) and locally focused culling (winter/spring) in the same time period.

Three groups of deer were specified as being part of the CWD surveillance process: the (1) *total population*, (2) *testable subpopulation*, and (3) *tested group*. The *total population* was the largest group and represented the entire cervid herd for which the true prevalence is of interest, with that herd including up to 20,000 deer. This *total population* size makes results applicable to spatial extents as large as a county in Illinois [16]. The *testable subpopulation* was the subset of the total deer population that was harvested and therefore had the potential to be tested for CWD. The *tested group* was the subset of the *testable subpopulation* that was actually tested for CWD using immunohistochemistry (IHC) and/or enzyme-linked immunosorbent assay (ELISA).

The size of the *testable subpopulation* was based on past deer mortality data from Illinois collected by the Illinois Department of Natural Resources (IDNR) between fiscal years 2003 and 2023 [16]. The 99^th^ quantile of the mortality data was 4,745 deer in a single county and fiscal year. Thus, 5,000 deer was set as the maximum *testable subpopulation* size, 100 deer was set as the minimum, and the interval between these values was divided into increments of 100 (ex. 100, 200 … 4,900, 5,000) to get the vector of potential *testable subpopulation* sizes. A vector was used rather than a distribution to allow simulation results to be tied to specific *testable subpopulation* sizes so that managers using the output table could find the number of harvested deer they have available and identify the associated testing requirements. We chose 100 deer as the minimum testable subpopulation size because we wanted that group’s apparent prevalence to be within ±2% of the population’s true prevalence and the fewest number of infected deer that could yield an apparent prevalence with that margin of error without involving a large truncation of the lower bound was 2 (0.000001% ≤apparent prevalence ≤ 4%).

The *total population* was defined as the *testable subpopulation* multiplied by 4, which was based on previous studies that reported an annual mortality rate for white-tailed female deer of ~25% [17,18]. In Illinois, roughly the same percentage of females and males are harvested every year, and so this rate for females was extrapolated to the entire population [19]. The choice to model the population as finite allowed for consideration of the potential effect of herd size on simulation outcomes.

*Tested group* sizes ranged in value from 10 deer to the size of the *testable subpopulation* in increments of 10 (ex. 10, 20 … 4,990, 5,000 for a *testable subpopulation* size of 5,000). The true prevalences of CWD examined in this simulation ranged from 0.5% to 20% in intervals of 0.5% (ex. 0.5%, 1% … 19.5%, 20%). All combinations of true prevalence, *testable subpopulation size*, and *tested group* size were assessed by this simulation.

Diagnostic test sensitivity was also incorporated into the model. This accounted for imperfections in laboratory results due to difficulties with identifying recently infected deer because prion accumulation in the diagnostic tissues is slow [20]. The outcome of whether (1) or not (0) the diagnostic test correctly reflected the deer’s true disease status was characterized as a random binomial distribution whose probability of producing the deer’s true disease status was equal to the sensitivity of an enzyme-linked immunosorbent assay (ELISA) test for CWD, which is often used as a first-line screening for diagnostic tissues before confirmation via immunohistochemistry (IHC) [20]. The sensitivity used as the probability of success in the random binomial distribution was generated by a random uniform distribution bounded by 0.92 and 0.99 [20], with this range obtained from field studies of test sensitivity in lymph nodes [20,21]. It was assumed that there were no false positives.

Once parameterized, the simulation was run for all combinations of true prevalence, *total population* size, *testable subpopulation* size, and *tested group* size for a predetermined number of iterations (see section *‘Determining number of simulation iterations’)*. The first step of the simulation was to generate a *total population* of deer with a certain true prevalence, with each deer defined by their CWD status of positive (1) or negative (0). This *total population* was then randomly sampled without replacement to get a *testable subpopulation* of the specified size. The deer in the *testable subpopulation* were then randomly sampled without replacement to create the *tested group* of the specified size. Next, a diagnostic test outcome for each tested deer was generated. This was done by sampling from the random uniform distribution of test sensitivities (0.92 to 0.99) to get a vector equal in length to the *tested group* size, then using each of those sensitivities as the probability of success in a random binomial distribution to get a vector of 0’s and 1’s of the same length. Each deer was assigned their respective diagnostic test outcome, with a 1 indicating the test correctly identified that deer’s disease status and a 0 indicating an incorrect test result.

**Figure 1.**
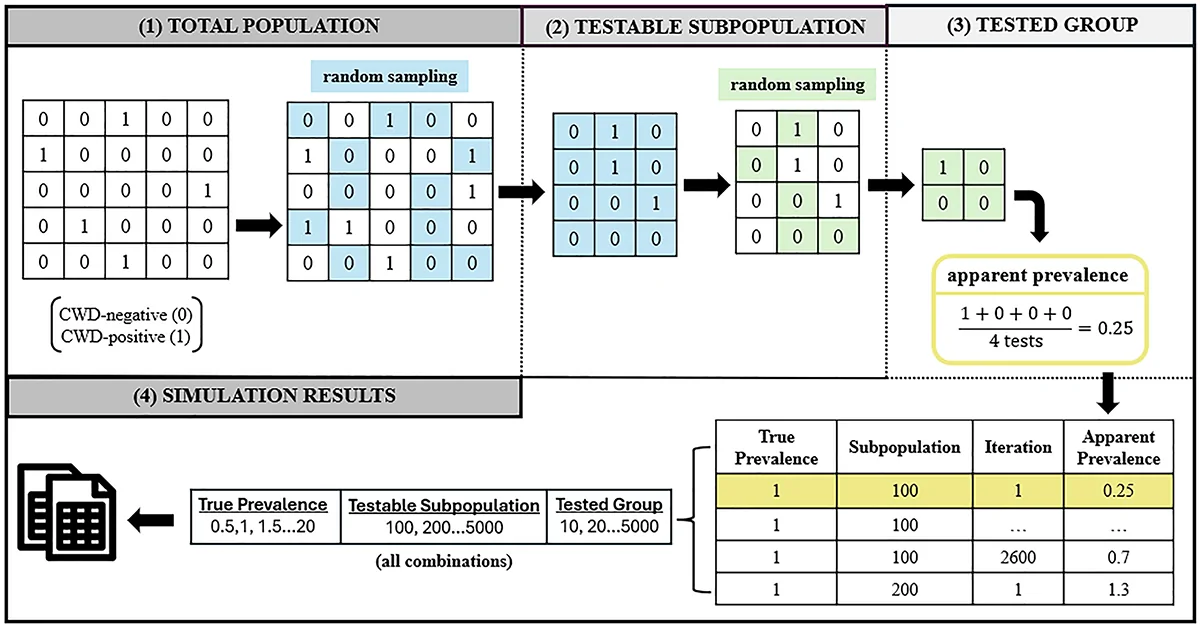
Flowchart of the Monte Carlo simulation of the number of deer that need to be tested for chronic wasting disease (CWD) (*tested group*) out of a subset of the *total population* (*testable subpopulation*) with CWD-positive (1) and CWD-negative (0) deer to accurately approximate the true prevalence using apparent prevalence with a ± 2% margin of error.

To get the apparent prevalence, the CWD statuses of the tested group were added together and divided by the size of the *tested group*. This apparent prevalence was the final output for a single iteration of the Monte Carlo simulation. Figure 1 visualizes these simulation steps. All code for this simulation is available at DOI: https://doi.org/10.13012/B2IDB-5278362_V2.

### Model justification

The Monte Carlo simulation of testing requirements for CWD surveillance utilizes standard random sampling at all three stages of the model to represent how the deer tested for CWD are not directly from the *total population* but rather are from the subset of the *total population* that hunters harvest and allow to be tested. This omits heterogeneity in deer distribution on the landscape, as well as spatial clustering of CWD cases, but we believe these omissions are justified because of two realities of hunting: (1) hunters can only successfully harvest deer in areas where deer are present and (2) hunters can only harvest the deer they encounter by random chance. This first reality implicitly accounts for differences in deer presence on the landscape, with hunters favouring the same locations as deer, so long as they have land access. The second reality means that the inclusion of the transition between the *total population* and *testable subpopulation* bypasses the need to quantify complex parameters like changing population dynamics, animal contact rates, or disease susceptibility because those factors do not affect a hunter’s chance of encountering any one deer in a particular area at a particular time. In short, this model utilizes random sampling because the deer a hunter comes across can be considered a random sample of the local population.

It is true that, depending on the population of interest, our estimates may over or underestimate the ‘perfect’ minimum number of deer to test for CWD. However, since there does not yet exist an easy-to-use reference table for managers containing estimates based on stochastic modelling of the CWD surveillance process, it was decided that such simplification was appropriate to achieve our goal.

### Determining number of simulation iterations

To determine how many iterations were needed for the Monte Carlo simulation to converge, the simulation was run for three true prevalences − 1%, 10%, and 20% - with a *testable subpopulation* size of 100 and a *tested group* size of 10. These three parameter combinations were run 20 times each for 10,000 iterations. All 60 simulation outputs were then examined to find the critical number of iterations above which the cumulative apparent prevalence at a given iteration did not differ by more than 0.5% from the true prevalence for all 10,000 iterations. This threshold of 0.5% was chosen because an error larger than this would result in rounding to the wrong apparent prevalence of those examined.

### Chronic wasting disease (CWD) testing simulation results

Once the appropriate number of iterations for the simulation was identified, the simulation was run with all combinations of true prevalence, *testable subpopulation* size, and *tested group* size for the determined number of iterations per combination.

Two criteria, ‘accuracy’ and ‘detection’, were used to determine whether each combination of *testable subpopulation* size and *tested group* size (i.e., 150 tests from a *testable subpopulation* of 600 deer) yielded an accurate approximation of the associated true prevalence. *Detection* was defined as the percentage of simulation iterations that included ≥ 1 CWD-infected deer in the *tested group* (apparent prevalence > 0). A minimum of 95% detection was required to reduce, but not eliminate, the chance of falsely reporting an absence of disease. *Accuracy* was defined as the percentage of simulation iterations that yielded an apparent prevalence within ±2% of the *total population’s* true prevalence. At least 95% of simulation iterations had to meet this criterion for that *tested group* size to be considered large enough to accurately approximate the true prevalence. The final reference table for managers included the number of deer to test out of those harvested by hunters from a CWD-affected population to detect the disease with an accuracy of ±2% (DOI: https://doi.org/10.13012/B2IDB-5278362_V2).

### Comparison to standard sample size equation

The results of the Monte Carlo simulation were compared to those from standard sample size calculations using *Equations 1* and *2* [22]. Z was the z-score of the desired 95% confidence interval (1.96), while precision was set equal to 0.02 (2%).

$$n_{0}=\frac{Z^{2}*\mathrm{trueprevalence}*\left(1-\mathrm{trueprevalence}\right)}{\mathrm{precisio}n^{2}}$$

$$N=\frac{n_{0}}{1+\frac{n_{0}-1}{\mathrm{totalpopulation}}}$$

### Comparison of simulation results to CWD surveillance in Illinois

The IDNR has conducted management and surveillance for CWD since it was first detected in northern Illinois in fiscal year 2003 [23]. We compared the simulation results to CWD surveillance data collected between fiscal years 2003 and 2023 [23] to determine if enough deer were tested for CWD to be confident that the calculated apparent prevalence was an accurate approximation of the true prevalence. This was relative to the *testable subpopulation* size, which was set to be equal to the total number of hunter-harvested deer in a county and fiscal year [16]. Two metrics were employed for this analysis. The first metric was the percentage of county/fiscal year combinations that tested enough deer for CWD to confidently detect the population’s true prevalence. Second, the lowest true prevalence accurately detected for any county/fiscal year was also calculated to assess the feasibility of our guidelines.

## Results

### Simulation convergence

**Figure 2.**
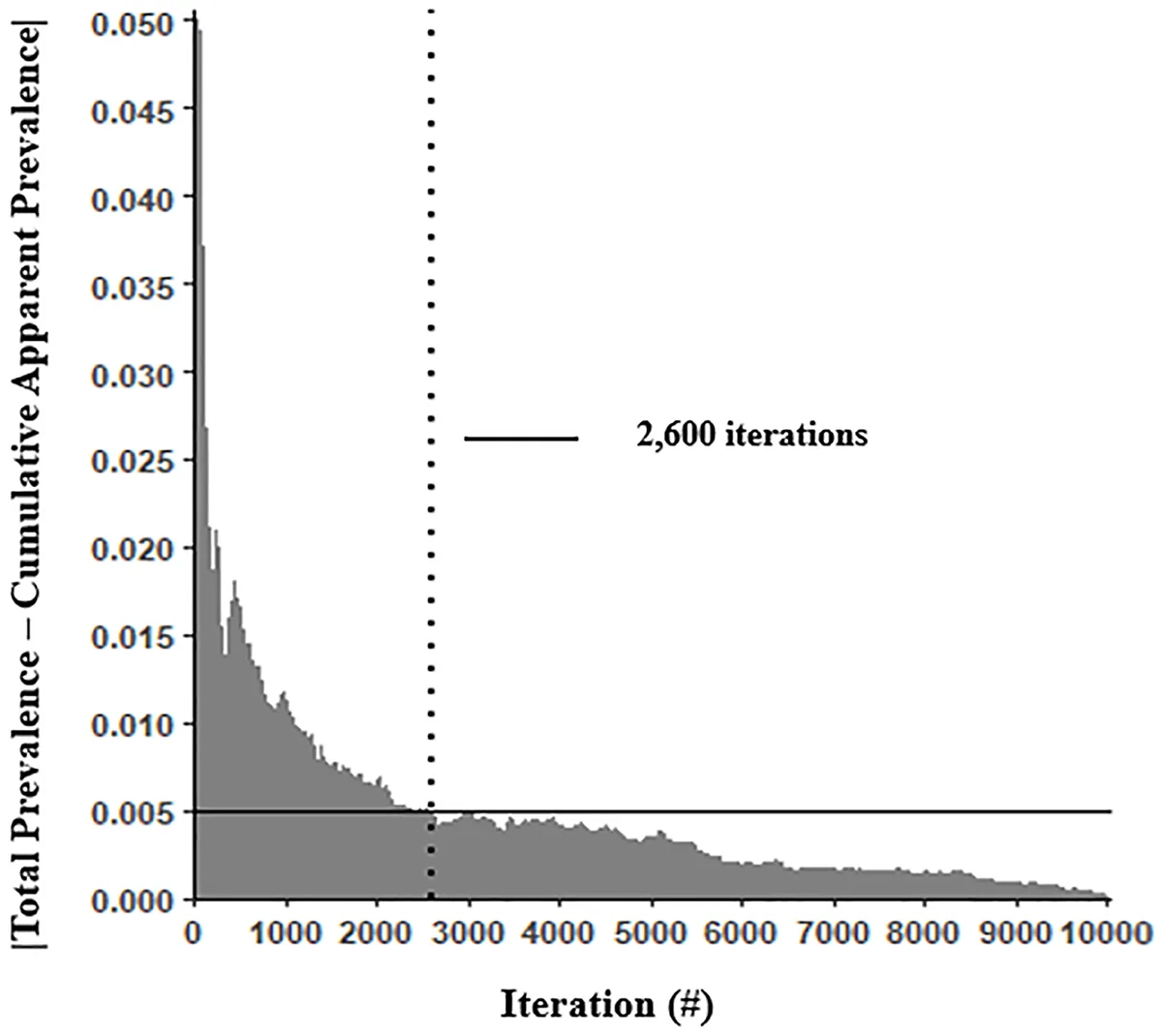
Convergence plot showing the difference between the true prevalence and cumulative apparent prevalences (y-axis) at each iteration (x-axis). The solid horizontal line represents the threshold of 0.5%, which was used to determine the level of accuracy needed for rounding to be correct. The dotted vertical line shows the necessary number of iterations to reach this accuracy.

Convergence of the simulation for a *testable subpopulation* size of 100, *tested group* size of 10, and true prevalences of 1%, 10%, and 20% were plotted together in Figure 2 as the difference between the true prevalence and cumulative apparent prevalences (y-axis) at each iteration (x-axis). Examination of Figure 2 led to 2,600 iterations being selected for all subsequent analyses.

### Chronic wasting disease (CWD) testing simulation results

Table 1 provides a small subset of the full reference table showing the minimum number of deer that need to be tested for CWD to detect each of the modelled true prevalences (0.5–20%) with a margin of error of ±2% in ≥95% of simulation runs. These testing guidelines are compared to the sample size estimations obtained from standard equations (*Equations 1* and *2*). The minimum *testable subpopulation* size is the fewest number of deer that must be available for testing to be able to detect the given true prevalence using the specified number of tests. For example, based on Table 1 it can be said that to detect a true prevalence of CWD of 0.5%, at least 5,000 deer need to be harvested by hunters and at least 600 of those deer must be tested for CWD to obtain an apparent prevalence between 0.000001% and 2.5%. The full reference table is provided at DOI: https://doi.org/10.13012/B2IDB-5278362_V2.

**Table 1.** Minimum number of deer to test for chronic wasting disease (CWD) based on Monte Carlo simulation results, compared to the suggested sample size from a standard equation (*equations 1* and *2*) for the tested true prevalences. Each number of tests corresponds to the sample size needed to obtain an apparent prevalence ±2% the *total population’s* true prevalence with 95% confidence. The minimum *testable subpopulation* size reflects how many deer must be available for sampling out of the *total population* for testing to detect that true prevalence. The *total population* is equal to 4 times the *testable subpopulation*.

True Prevalence (%)	Minimum Testable Subpopulation Size (# deer)	Minimum Tests (# deer)	
		Simulation	Standard Equation
0.5	5000	600	48
1	300	280	88
2	200	150	153
3	200	180	207
4	300	250	282
5	400	330	355
6	400	390	405
7	600	530	496
8	600	550	546
9	700	660	614
10	800	750	681
11	900	890	746
12	1000	950	809
13	1100	1080	871
14	1200	1190	932
15	1400	1360	1005
16	1500	1450	1062
17	1700	1640	1130
18	2100	1880	1213
19	2200	2140	1266
20	2500	2430	1310

### Comparison of simulation results to CWD surveillance in Illinois, MarkovUSA

Comparing the output of this sample size simulation to the CWD surveillance conducted by the IDNR in Illinois between fiscal years 2003 and 2023 showed that 16.14% of counties tested at or above the levels suggested by the Monte Carlo simulation. When testing was sufficient, IDNR surveillance efforts were able to detect true prevalences as low as 1%, demonstrating that the existing surveillance can be effective at detecting lower levels of disease.

## Discussion

This study developed sample size recommendations for wildlife managers for detecting CWD in wild cervids using Monte Carlo simulation techniques as an improvement on standard sample size equations that can function as a stopgap until more detailed models can be created for the geographical area of interest. The contents of Table 1 show that, except for true prevalences of 0.5 and 1%, both the *testable subpopulation* and *tested group* sizes increase as the true prevalence increases, with ≥280 tests needed from 300 harvested deer to detect a 1% true prevalence and ≥2,430 tests needed for 2,500 harvested deer to detect a 20% true prevalence. This increasing trend is due to the constraints put on the model outputs, which required a ≥ 95% detection rate and a margin of error of ±2% in at least 95% of simulation runs. These constraints are harder to meet as the true prevalence increases, and this necessitates larger *testable subpopulation* sizes. A parallel trend is observed in the sample sizes suggested by the standard equation (Table 1).

When comparing the standard equation to the simulation, it can be seen that the majority of sample sizes advised by the simulation are higher than those from the standard equations. This was expected, since the simulation accounts for uncertainty, while the standard equations do not, which emphasizes the importance of including stochasticity in models of CWD surveillance to avoid underestimating testing needs and overestimating confidence in surveillance statistics. However, this stochasticity also means that following the guidelines in the reference table will not yield exactly the same results every time, but the accuracy criteria employed increase the likelihood that the outcome will be close. The reference table should function as a minimum surveillance target, and efforts should always be made to test as many deer as possible.

We also documented the challenges of detecting CWD faced by the IDNR and those challenges may impact their CWD management program. Analysis of surveillance data collected between fiscal years 2003 and 2023 in the 19 counties that detected at least one CWD case within that time frame found that 16.14% of efforts tested enough deer for their estimated apparent prevalence to accurately approximate the true prevalence, though some efforts were able to detect true prevalences as low as 1%. This is important because it shows that surveillance can obtain sufficiently large sample sizes with enough hunter participation to detect lower prevalences, which is often a concern with disease surveillance, especially in free-ranging wildlife. These results emphasize how essential hunter cooperation is for state agencies trying to manage wildlife diseases and show that the sample sizes recommended by the model are achievable.

As all models do, our Monte Carlo simulation has some limitations. In this simulation, we only use *testable subpopulation* sizes up to 5,000 deer and *total population* sizes up to 20,000 deer, which means that these findings are not applicable to locations with higher hunter-harvest numbers. We also do not evaluate true prevalences above 20%, and so these tables would not be accurate for locations experiencing higher disease burdens. Another major limitation of this simulation is that it is designed for the randomness involved with hunter-harvest and is therefore likely to overestimate the amount of testing required for managers doing more targeted sampling. In those situations where targeted sampling is occurring, it would be better to use estimates obtained from more complex models – if they exist – to set less conservative sampling goals.

Our assumption of random sampling by hunters also ignores the potential role of hunter bias in deer selection if hunters can choose from multiple deer. This highlights an area of future work where the simulation could be expanded to further broaden its usefulness. Additional areas of exploration include applying our methods to other deer diseases, such as epizootic haemorrhagic disease (EHD) or bovine tuberculosis (BTV) [24], or to other wildlife species, as well as adding considerations such as deer habitat [25].

## Data Availability

All data used in the analyses of this study can be viewed and downloaded from the Illinois Data Bank (https://doi.org/10.13012/B2IDB-5278362_V1).
